# Diseases of the Gall-Bladder and Bile-Ducts

**Published:** 1900-09

**Authors:** 


DISEASES OF THE GALL-BLADDER AND BILE-DUCTS.—By A. W. Mays Robson, F. R. C. S., Senior Surgeon to the General Infirmary at Leeds; Emeritus Professor of Surgery in the Yorkshire College of the Victoria University; Member of Council, and Hunterian Professor of Surgery and Pathology at the Royal College of Surgeons of England. Assisted by Farquar Macrae, M. B., C. M. (Glas.). Second Edition. Pp. 313. Price, ——. New York: William Wood Company. 1900.

The book is a British production and gives an elaborate description of the gall-bladder and bile-ducts, the diseases to which they are subject and the operations and treatment best adopted for relief. The operation proposed for gall-stone has given the author a mortality of only 1.7 per cent., under the following proposition: "That as soon as gall-stones give serious trouble, their removal by operation is the most rational method of treatment." This is a most excellent work, and should find ready sale in every town and hamlet where surgery of the gall-bladder and bile-duct is done.

